# Feasibility Study of Applying Modified Nano-SiO_2_ Hyperbranched Copolymers for Enhanced Oil Recovery in Low-Mid Permeability Reservoirs

**DOI:** 10.3390/polym11091483

**Published:** 2019-09-11

**Authors:** Nanjun Lai, Lei Tang, Na Jia, Dongyu Qiao, Jianlin Chen, Yong Wang, Xubin Zhao

**Affiliations:** 1 School of Chemistry and Chemical Engineering of Southwest Petroleum University, Chengdu 610500, China; 201811000045@stu.swpu.edu.cn; 2 State Key Laboratory of Oil and Gas Reservoir Geology and Exploitation, Chengdu University of Technology, Chengdu 610500, China; 3 State Key Laboratory of Molecular Engineering of Polymer, Fudan University, Shanghai 200433, China; 4 Faculty of Engineering and Applied Science, University of Regina, Regina, SK S4S 0A2, Canada; Na.Jia@uregina.ca; 5 Engineer Technology Research Institute, CNPC Xibu Drilling Engineering Company Limited, Urumqi 830001, China; xjqdy@cnpc.com.cn; 6 CNPC Xinjiang Oilfield Company, Karamay 834000, China; 201811000047@stu.swpu.edu.cn (J.C.); lainanjun@swpu.edu.cn (Y.W.); lainanjun@163.com (X.Z.)

**Keywords:** modified nano-SiO_2_, hyperbranched copolymer, enhanced oil recovery, low-mid permeability reservoir

## Abstract

To improve oil recovery significantly in low-mid permeability reservoirs, a novel modified nano-SiO_2_ hyperbranched copolymer (HPBS), consisting of polyacrylamide as hydrophilic branched chains and modified nano-SiO_2_ as the core, was synthesized via an in situ free radical polymerization reaction. The structure and properties of the hyperbranched copolymer were characterized through a range of experiments, which showed that HBPS copolymers have better stability and enhanced oil recovery (EOR) capacity and also smaller hydrodynamic radius in comparison with hydrolyzed polyacrylamide (HPAM). The flooding experiments indicated that when a 1000 mg/L HPBS solution was injected, the resistance factor (RF) and residual resistance factor (RRF) increased after the injection. Following a 98% water cut after preliminary water flooding, 0.3 pore volume (PV) and 1000 mg/L HPBS solution flooding and extended water flooding (EWF) can further increase the oil recovery by 18.74% in comparison with 8.12% oil recovery when using HPAM. In this study, one can recognize that polymer flooding would be applicable in low-mid permeability reservoirs.

## 1. Introduction

The exploration and exploitation of unconventional resources is an inevitability due to the pace and trend of the development of the economy. Low-mid permeability reservoirs are one of the most important unconventional resources with both an enormous economic value and an abundant quantity of reserves [1,2,3]. The classification of reservoirs is shown in Table 1 [4].

These types of unconventional reservoirs possess a plethora of distinctive features, which include small pore throats, high specific surface area, severe heterogeneity and presence of low viscosity crude oil [5,6,7]. For these reasons, some researchers have studied gas and water injection to restore reservoir energy for enhanced oil recovery (EOR). For example, Jakobsson [8] found that deep migration of injected hydrocarbon gas could broaden the sweep efficiency at the Ekofisk reservoir (K = 0.1 × 10^−3^ μm^2^ ~ 5 × 10^−3^ μm^2^). Reservoir simulation indicated that gas injection could recover an increased 2 to 3% of original oil in place (OOIP). Liu [9] reported that 78 oil wells have been put into production by injecting water in Block BAO14 at Liaohe Oilfield, (K = 27.0 × 10^−3^ μm^2^) with oil production of 289 metric tons/day, and it resulted in an additional 7.76% oil recovery. In summary, efficient enhanced oil recovery is challenging because of the issues present in low-mid permeability reservoirs due to large amounts of trapped oil after gas and water flooding [10].

Gas and water flooding are two commonly used secondary recovery methods in the industry; nevertheless, usually more than 60% of the original oil in place will still be left after the application these techniques [11,12]. Polymer flooding is a commercial technology that has been extensively utilized as an EOR method for high permeability reservoirs [13,14,15]. The polymer can increase the viscosity of the water phase, decrease the mobility ratio between the water and oil phases and, thus, increase the sweep efficiency, compared with water flooding [16,17]. According to the bridge principle [18], when the ratio of the pore throat radius to the gyration radius (*R_g_*) of a polymer molecule is greater than 5, the polymer will pass through the pore throat [19]. Hydrolyzed polyacrylamide (HPAM) and bio-polymers are two common types of polymers that are currently utilized in polymer flooding [20,21]. Their average hydrodynamic radius is approximately 4~16 µm, which is not compatible with the pore throat sizes of low-mid permeability reservoirs (10^−2^~10 µm) [22]. In addition, the efficient application of polymer flooding is restricted by shear degradation. During polymer flooding, the polymer’s molecular chains will deteriorate due to shear degradation while travelling through the mixer, pipeline, pump, perforations and porous medium. Hence, there is a significant reduction in the viscosity of the polymer solution [11]. Moreover, the importance of the effect on the polymer viscosity is highlighted due to the comparatively diminutive pore throat of the low-mid permeability reservoir [23,24,25].

Gao [26] reported that polymers with highly branched molecular architectures have low viscosity and numerous highly functional end groups, making them ideal as a flooding polymer in a low-mid permeability reservoir. Petreska [27] showed that the composition of the block copolymers, as well as the thermal treatments of the copolymers, greatly influenced the viscoelastic properties of the block copolymers. Linul [28] showed that by adding 1.5 wt.% aluminum microfibers (AMs) into the polyurethane (PU) foam matrix, the compressive strength and energy absorption performances of the matrix would be significantly enhanced. Marsavina [29] examined the effect of glass fiber reinforcements on the mechanical and morphological properties of polyurethane rigid foams. With the increase in glass fiber mass content, the mechanical properties of the foams could significantly increase to 121% in stiffness and 101% in strength.

For the reasons stated above, this research aimed to synthesize a hyperbranched copolymer based on modified nano-SiO_2_ for enhanced oil recovery in low-mid permeability reservoirs. The polymer has outstanding physical and mechanical properties, and also a suitable hydrodynamic radius for injection within low-mid permeability reservoirs [30,31].

## 2. Experimental Section

### 2.1. Experimental Materials

Analytical grade acrylic acid (AA), acrylamide (AM), maleic anhydride (MA), *N*,*N*-dimethylformamide (DMF), toluene, sodium bisulfite, ammonium persulfate and ethanol were purchased from Chengdu Kelong Chemical Reagent Co., Ltd., Chengdu City, China. These chemicals were used without further purification. Furthermore, the nano-SiO_2_ (15 nm), and 3-aminopropyltrimethoxysilane (KH540) were purchased from Shanghai Aladdin Chemical Reagent Co., Ltd., Shanghai City, China. Water was double deionized with a Millipore Milli-Q system to produce the 18 MΩ cm deionized water.

### 2.2. Preparation of Hyperbranched Copolymers (HBPSs)

**Figure 1:**
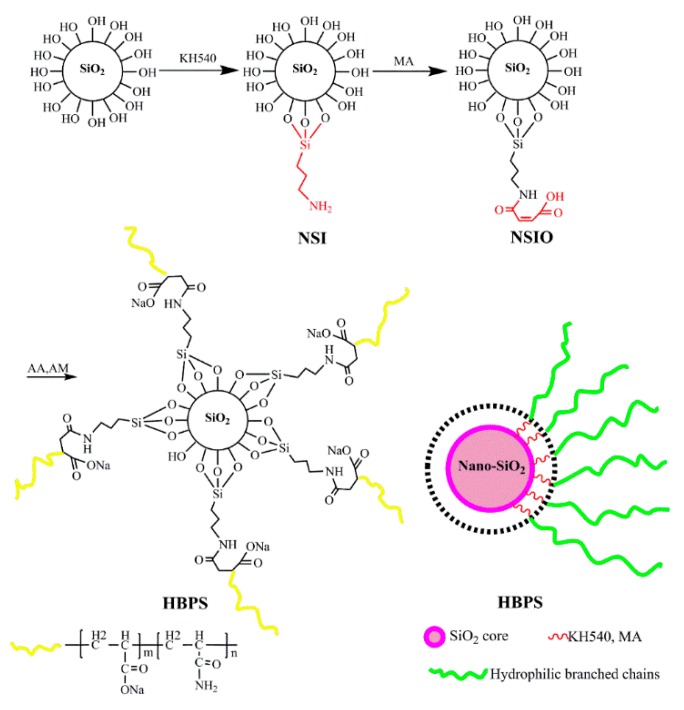
Synthesis schematic route of NSIO and HBPSs.

Modified nano-SiO_2_ hyperbranched copolymer, consisting of polyacrylamide as hydrophilic branched chains and modified nano-SiO_2_ as a core, was synthesized through an in situ free radical polymerization reaction. Firstly, 5.0 g nano-SiO_2_ and 100.0 mL toluene solvent were combined in a 250 mL round flask. Then different amounts of KH540 were slowly added into a round flask, and the resultant mixture was stirred at 80 °C for 12 h. Afterwards, ethanol was repeatedly utilized to wash away the unreacted KH540 and toluene solvent in a rotary steaming instrument. The modified nano-SiO_2_ (NSI) white powder product was formed after drying the mixture at vacuum conditions. Secondly, 20.0 mL *N*,*N*-dimethylformamide (DMF) was added into a wide neck flask as a solvent to dissolve 1.0 g maleic anhydride (MA). Another 20.0 mL DMF was added into a new wide neck flask as a solvent to dissolve 1.0 g NSI, and then the NSI solution was slowly added into the MA solution. After the addition, the mixture was stirred at 70 °C for 12 h. Following that, the alcohol and deionized water were repeatedly utilized to wash the modified product. Next, the product was vacuum dried to get the functional monomer of nano-SiO_2_ (NSIO) at different degrees of modification by controlling the amount of KH540. Furthermore, functional monomers of NSIO, AA and AM were dissolved in distilled water inside a 100 mL wide neck flask with a specified concentration (the AM:AA ratio was 7:3). The pH of the solution was adjusted to 7.4, and the solution was kept in a water bath at 40 °C. The initiator (the n (NaHSO_3_): n ((NH_4_)_2_S_2_O_8_) ratio was 1:1) was slowly added into the wide neck flask using a pipette. The polymerization process was sustained for 4–6 h, and HBPSs were synthesized. The conditions of copolymerization reactions are presented in Table 2. The final HBPSs were obtained after washing, pulverization and drying, and HBPS-1 and HBPS-2 were synthesized using identical experimental and purification methods as described above. HBPS-1 is the product in which 26% hydroxyl groups on the surface of nano-SiO_2_ were modified by KH540, and HBPS-2 is the product in which 48% hydroxyl groups on the surface of nano-SiO_2_ were modified by KH540. The method of determining the content of hydroxyl groups is provided in the Supplementary Materials (Quantity of hydroxyl groups by CH_3_MgCl titration is shown in Table S1 by using the flow-process of Figure S1, content of surface amino groups after grating KH540 to nano-SiO_2_ is shown Table S2, effect of KH540 Dosage on surface modification degree of nano-SiO_2_ is shown in Figure S2). The scheme of the synthesis route is shown in Figure 1.

### 2.3. Characterization

The infrared (IR) spectra analysis on KBr pellets was conducted using a WQF-520 infrared spectrometer, which was purchased from Beijing Rayleigh Analytical Instrument Co., Ltd., Beijing City China. NSIO, HBPS-1 and HBPS-2 were characterized using IR. The ^1^H-NMR analysis was performed using a Bruker AC-E 200 NMR spectrometer (Bruker Daltonics Inc., Billerica City, Massachusetts, USA) with D_2_O solvent. The hydrodynamic radius (*R_h_*) distribution, weight-average molecular weight (*MW*) and gyration radius (*R_g_*) of each polymer in deionized water were carried out using a BI-200SM Dynamic/Static Laser Light Scattering that was purchased from the Brookhaven Instruments Corporation, Austin City, Texas, USA. The morphology of each sample in deionized water was observed using a Quanta 450 Environmental Scanning Electron Microscope that was purchased from the FEI Company, Hillsboro City, Oregon, USA.

### 2.4. Stability Experiments

The stability of HPAM, HBPS-1 and HBPS-2 was evaluated to examine the effects of temperature, salt content and mechanical shearing strength on the polymer solutions. Apparent viscosity was measured with the Brookfield DV-III Programmable Rheometer (Brookfield Co., Middleboro City, Massachusetts, USA). A brine of 5727 mg/L total dissolved solids (TDS) was used to prepare the polymer solution with a concentration of 1000 mg/L, and the ionic composition is shown in Table 3 (the ionic concentration of the brine was measured by Shengli Oilfield, Dongying City, Shandong Province, China). Apparent viscosity of the polymer solution at different temperatures, salinities and mechanical shear strengths was measured. Eventually, the influence of mechanical shear strength on apparent viscosity was investigated using a WT-VSA2000B Waring Mixer (Beijing Exploration Engineering Research Institute of China Geological Survey) at a shear strength of 3600, 7200 and 11,000 RPM for 20 s.

### 2.5. Viscoelasticity Tests

A polymer with excellent viscoelasticity has a strong ability to pull small, trapped oil drops out of the porous media. Therefore, good viscoelasticity obviously is a factor that can improve the displacement efficiency [32]. A HAAKE MARS RS600 rheometer (HAAKE Technik Co., Vreden City, Germany) was utilized to assess the viscoelasticity of 1000 mg/L solutions of HPAM, HBPS-1 and HBPS-2 at 70 °C. It uses a DG41Ti measurement system with a scan frequency of 0.1~10 Hz. The brine was utilized to prepare the original polymer solution with a concentration of 5000 mg/L. The original polymer solution was diluted into the solutions of 1000 mg/L. The relationships between the viscous/elastic moduli and scanning stress of polymer dilute solutions were examined.

### 2.6. Sand Pack Model Flooding Tests

Further equipment, procedures and permeability measurement details can be found in the references [33,34]. The sand pack model consists of an injection pump, a sand pack tube, a pressure recording system and gas/liquid collectors. The tube, with a length of 25 cm and an inner diameter of 2.5 cm, was filled with 140–160 unit mesh quartz dried sand, which was washed using a 15 wt.% hydrochloric acid solution, and then was rinsed using water until the pH reached 7. The filled tube was further dried to prepare for the tests. For each experiment, fresh sand was used to ensure consistent conditions. The pore volumes (PV) were determined through the mass difference of the sand pack tube before and after the brine saturation (TDS of 5727 mg/L; ionic composition is shown previously in Table 3). The dried sand packed tube was flooded with the brine at 0.5 mL/min, and the pressure difference was recorded to determine the permeability using Darcy’s law [35]. Both polymer injection and subsequent extended water flood were set at 0.5 mL/min [36]. The polymer solution of 1000 mg/L was utilized to investigate the feasibility of using a hyperbranched polymer to enhance oil recovery in low-mid permeability reservoirs. More specifically, the injection capability, mobility control and EOR performance of the application of the polymer was examined and compared. The injection capabilities of HBPS-1, HBPS-2 and HPAM in low-mid permeability reservoirs were measured, and the mobility control capability of the polymer was evaluated by analyzing the resistance factor (RF) and residual resistance factor (RRF) (shown in Equations (1) and (2)) [10]. The crude oil that was collected from Shengli Oilfield in China had a viscosity of 5.3 mPa·s at 70 °C and a shear rate of 7.34 s^−1^. To prepare for the flooding test, the model was initially heated inside an oven at 70 °C, then crude oil was continuously injected into the model at 0.5 mL/min until no more water was produced from the effluent pipe, which indicates that the water saturation had reached the connate water saturation. Then, the model was kept in the oven for another 48 h. Secondly, the brine was injected at 0.5 mL/min to displace oil until less than 2.0 vol.% of oil cut per sample was observed. Next, 0.3 PV of polymer solution was injected. Following polymer injection, the brine was injected continuously at 0.5 mL/min until less than 2.0 vol.% of oil cut per sample was obtained. The enhanced oil recovery factor (EOR) for polymer flooding was calculated using Equation (3) [37].

(1) $$RF=\frac{\lambda_{w}}{\lambda_{p}}=(\frac{K_{w}}{\mu_{w}})/(\frac{K_{p}}{\mu_{p}})$$

(2) $$RRF=\frac{K_{wb}}{K_{wa}}$$

where $K_{w}$ is the water phase permeability (μm^2^), $K_{p}$ is the polymer phase permeability (μm^2^), $\mu_{p}$ is the polymer solution viscosity (mPa·s), $\mu_{w}$ is the aqueous phase viscosity (mPa·s), $K_{wb}$ is the permeability of the aqueous phase before polymer flooding and $K_{wa}$ is the permeability of the aqueous phase after polymer flooding (where $K_{wb}$ = $K_{w}$).

(3) $$EOR=E_{T}\;-\;E_{W}$$

where *EOR* is the recovery factor of polymer flooding, vol.%; *E_T_* is the total oil recovery combining the preliminary water flooding, polymer flooding and extended water flooding, vol.% and *E_W_* is the oil recovery of the for preliminary water flooding plus extended water flooding, vol.%.

## 3. Results and Discussion

### 3.1. Structure and Morphology of Hyperbranched Copolymer

#### 3.1.1. IR Characterization

**Figure 2:**
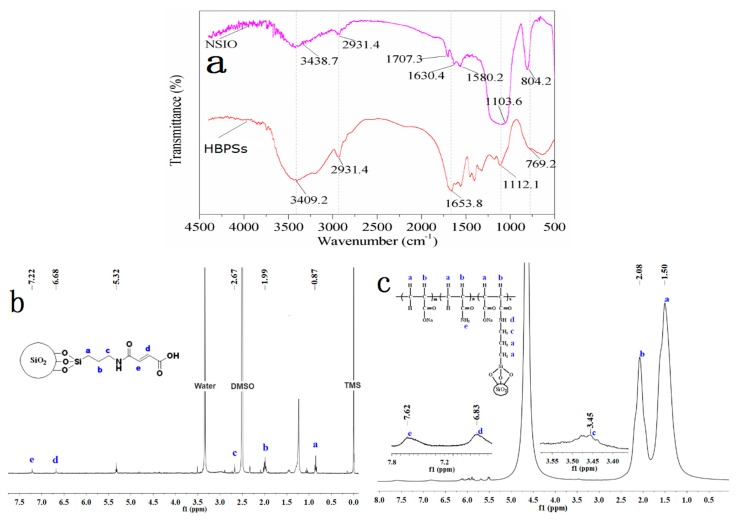
(**a**) IR spectrum, (**b**) ^1^H-NMR of NSIO and (**c**) ^1^H-NMR of HBPSs.

The chemical structure of monomer NSIO and copolymers HBPSs was confirmed using IR, and the results are shown in Figure 2a. The absorption at 3438.7, 2931.4, 1707.3 and 1630.4 cm^−1^ in NSIO indicated, respectively, that the existence of –NH–, –CH_2_–, C=O and C=C. All of these findings confirmed the existence of KH540 and MA on the surface of nano-SiO_2_. The characteristic peaks of HBPS was listed as follows: 3409.2 cm^−1^ (–OH stretching vibration), 3201.8 cm^−1^ (symmetric –NH_2_ stretching of amide group), 2931.4 cm^−1^ (–CH_2_– stretching vibration), 1653.8 cm^−1^ (C=O stretching vibration of amide group) and 1532.8 cm^−1^ (C=O stretching of carboxyl group). The results show the expected hyperbranched structure of the copolymers HBPSs.

#### 3.1.2. ^1^H-NMR Characterization

Figure 2b displays the ^1^H-NMR spectra of the NSIO functional monomer. The chemical shift values at 0.87, 1.99 and 2.67 ppm were attributed to the protons of –C**H**_2_–CH_2_–CH_2_–NH–, –CH_2_–C**H**_2_–CH_2_–NH– and –CH_2_–CH_2_–C***H***_2_–NH–, respectively. The signal observed peaks at 6.68 and 7.22 ppm were assigned to the protons of –CH=C**H**–COO– and –NHCO–C**H**=CH–. Figure 2c shows the ^1^H-NMR spectra of HBPS. The peak values at 1.50, 2.08, 3.45, 6.83 and 7.62 ppm were due to the protons of –C**H**_2_–, –CH_2_–C**H**(COONa)–, –C**H**_2_–NH–, –N**H**_2_ and –N**H**–, respectively. As can be seen from the ^1^H-NMR results, nano-SiO_2_ is copolymerized with AA and AM after being modified by KH540 and MA to prepare HBPSs.

#### 3.1.3. Diameter Distribution.

**Figure 3:**
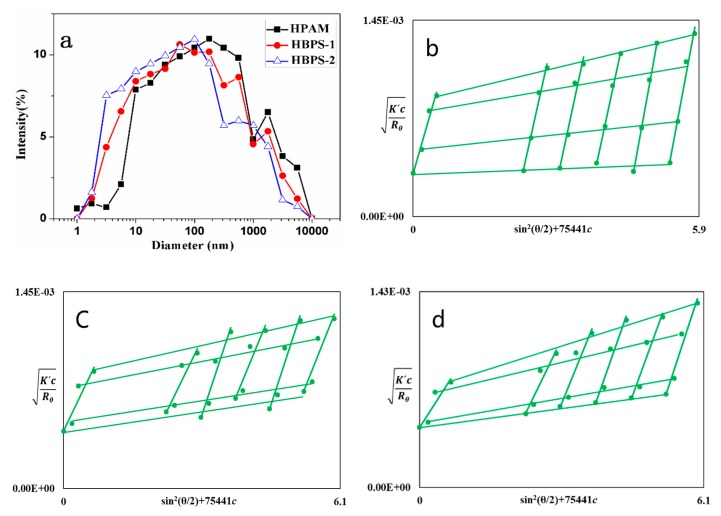
(**a**) Diameter distribution for HPAM, HBPS-1 and HBPS-2 with concentrations of 1000 mg/L, and Zimm plots of (**b**) HPAM, (**c**) HBPS-1 and (**d**) HBPS-2.

The production rate for low-mid permeability reservoirs is very low due to low permeability and porosity. There is a preconceived notion that polymer flooding cannot be applied to low-mid permeability reservoirs due to the incompatibility between the dimensions of the polymer molecules and the small pore throat sizes of the low-mid permeability reservoirs. Therefore, the molecular dimensions of the injected polymer must be small enough to meet the injection requirements of low-mid permeability reservoirs. The diameter distribution and Zimm plots of HPAM, HBPS-1 and HBPS-2 are shown in Figure 3a–d, and the principal structural parameters are shown in Table 6. The average hydrodynamic radius of the HBPS-1 and HBPS-2 molecules is observed to be 196 nm and 158 nm, respectively. These hydrodynamic radii are smaller than that of HPAM molecules (315 nm) with a similar *M*_W_ value. The significant difference in *R_h_* between the linear polymer HPAM and the hyperbranched copolymer HBPSs indicates that the multi-side-chain structure polymer can behave differently than those with the linear structure polymer. In addition, HBPS-2 was synthesized with a higher degree of modification of NSIO; thus, it has more branched chains. As a result, HBPS-2 has a shorter hydrodynamic radius than HBPS-1 at a similar MW value. As one can see in Figure 3, the displayed diameter distribution of HBPS-1 and HBPS-2 is broad compared with the HPAM diameter distribution. Consequently, the hydrodynamic radius of HPAM molecules is larger than those of HBPS-1 and HBPS-2, which poses a negative influence on the success of injection into the low-mid permeability reservoirs. Cao et al. [18] reported that a water-soluble polymer could be injected when the pore throat radius is larger than 5 times the *R_g_* of the polymer. According to the bridge principle and the experimental *R_h_* (196 nm) and *R_g_* (117 nm) values of HBPS-1, HBPS-1 can successfully be injected into a porous medium whose pore throat radius is larger than 585 nm. Similarly, the experimental *R_h_* and *R_g_* value of HBPS-2 (158 nm and 96 nm) indicates that HBPS-2 can be injected if the minimum pore throat radius is 480 nm. The unperturbed dimension (A) of the copolymer can be obtained by using Equations (4) and (5), and it shows the flexibility of the copolymer, as shown in Table 4. As the flexibility of the molecular chains increases, the number of possible conformations available under the same obstruction condition increases, and the number of molecular chains that would have been cut by the porous medium decreases. Therefore, when HPAM passes through the porous medium, its molecular chains are more readily destroyed when compared with the HBPSs.

The formulae to calculate the unperturbed dimension (A) and gyration radius (*R_g_*) of a polymer are [32]:

(4) $$R_{g}{}^{2}=\frac{1}{6}(h^{2}{)}_{0}$$

(5) $$A=\sqrt{(h^{2}{)}_{0}/MW}$$

where *A* is the unperturbed dimension of a polymer, nm (mol/g)^½^; ${\left(h^{2}\right)}_{0}$ is mean square end-to-end distance, nm^2^; *MW* is molecular weight of polymer, g/mol and $\left(R_{g}^{2}\right)$ is mean square gyration radius, nm^2^.

#### 3.1.4. Morphology

**Figure 4:**
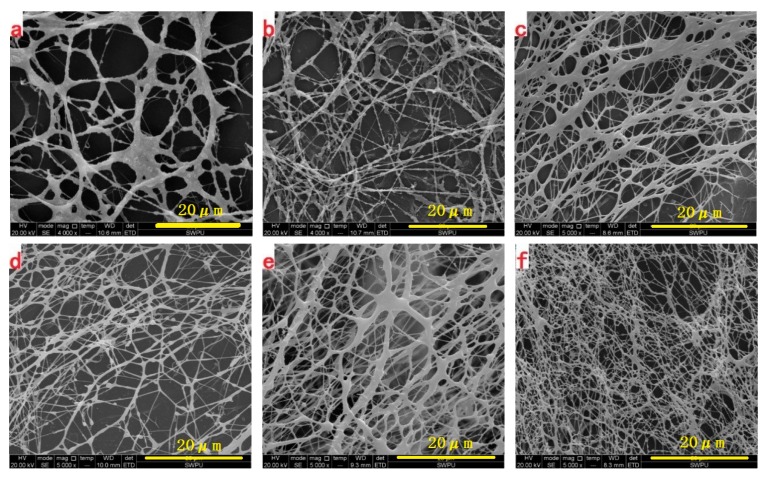
ESEM morphologies of (**a**,**b**) HPAM (before/after shearing), (**c**,**d**) HBPS-1(before/after shearing) and (**e**,**f**) HBPS-2 (before/after shearing) in deionized water (polymer concentration, 1000 mg/L).

To investigate the morphology of the synthesized polymers, ESEM was utilized to study the morphology of the unsheared and sheared hyperbranched HBPS-1/HBPS-2 copolymer and HPAM solutions at concentrations of 1000 mg/L. As can be seen in Figure 4, HBPSs are intertwined and a finer network structure is formed when compared with HPAM. A large number of HPAM molecular branches intertwine to form a network of structures before shearing, as shown in Figure 4a. After shearing, in Figure 4b, the accumulated HPAM molecular coils are destroyed and the coil size decreases. While for HBPS-1 and HBPS-2 solutions, a relatively even network structure is formed before shearing, as illustrated in Figure 4c,e. After shearing, although the molecular coils size reduces, the copolymer’s molecular coils are still intertwined and can reform into a finer network structure in Figure 4d,f. The network structure can prevent further degradation of the molecular chains. Comparing the microgram of copolymer HBPS-1 with HBPS-2, the network structure of the HBPS-2 solution is closer and more compact, and thus more shear resistant. This is due to the smaller hydrodynamic radius of HBPS-2, and the higher degree of modification during the synthesis process with nano-SiO_2_, which creates a larger number of branched chains to facilitate the acting force between polymer molecular chains.

### 3.2. Temperature Resistance

**Figure 5:**
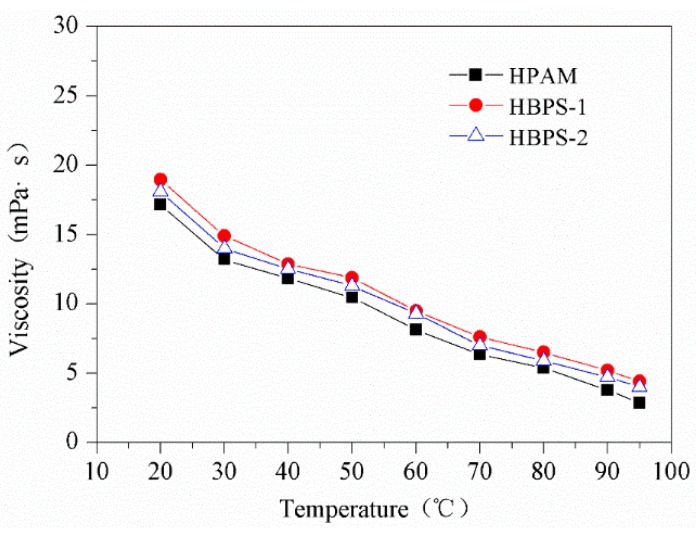
Effect of temperature on polymer viscosity.

It has long been acknowledged that temperature increase results in a viscosity reduction of the polymer solution. According to the analysis, the temperature has two-sided effects on the apparent viscosity of HBPSs copolymer solutions. Firstly, temperature rise augments the thermal degradation effects of the polymer molecules, the hydrodynamic radius becomes smaller and the viscosity is reduced. On the other hand, temperature rise increases the thermal motion of the polymer molecules. As a result, the contact probability between the polymer chains increases, which will help to form the polymer network structure in order to maintain the viscosity of the polymer solution. HBPS-2 has better stability than HBPS-1 because it contains more branched chains. In the case of HPAM, the temperature rise can accelerate the thermal degradation among the polymer molecules due to its linear structure, and the viscosity of HPAM is reduced significantly since HPAM does not have a hyperbranched structure. Although all three polymers’ viscosities are reduced by temperature increase, the viscosity of HBPSs is higher under the same conditions compared with HPAM. The results are illustrated in Figure 5.

### 3.3. Salt Resistance

**Figure 6:**
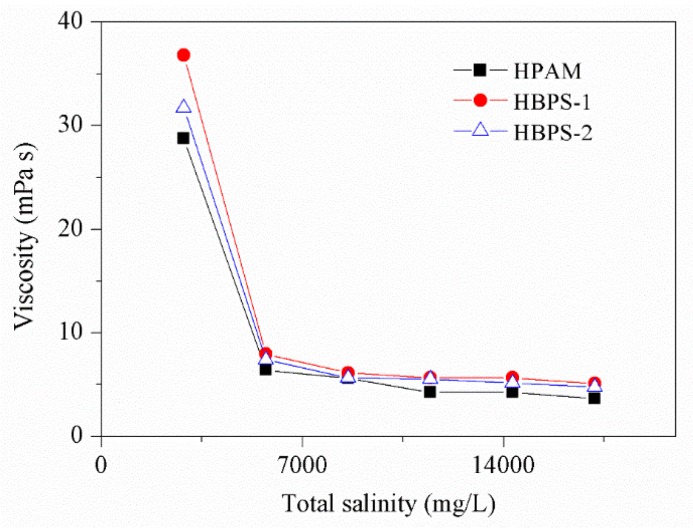
Effect of salinity on polymer viscosity.

The viscosity of all the polymer samples decreases significantly with increasing salt concentration, and then eventually stabilizes. Our analysis indicates that the charges in the polymer molecule chain can be shielded after the addition of a small amount of electrolyte molecules. Thus, the electrostatic repulsion between the polymer chains is attenuated [38], and polymer molecular chains will coil; then the viscosity of polymer solution decreases. When the dosage of additional electrolytes is increased to a certain level, shielding effect reaches its maximum, the curling of polymer molecular chains ceases and the viscosity reduction of polymer solution stops. Compared with HPAM, HBPSs copolymer has a hyperbranched network structure, which weakens the shielding effect of the electrolyte; therefore, it can reduce the coiling of the molecular chains and improve the copolymer’s salt resistance. In addition, the structure of hyperbranched HBPSs copolymer is more regular and predictable than HPAM. Salt causes a decrease in the coiling of the copolymer’s molecular chains, which is made to maintain the hydrodynamic radius of the copolymer. Therefore, the hyperbranched HBPSs copolymer solution has a higher viscosity under the same conditions compared with HPAM, as shown in Figure 6.

### 3.4. Mechanical Shearing Resistance

In the injection and flooding process, the polymer molecular chains are often damaged when exposed to shear from a mixer, pipeline, pump, perforation and porous medium. Therefore, the viscosity of the polymer solution will be significantly reduced [39,40]. In this study, the effects of mechanical shear strength are examined by using the Mixing Speed Governor (WT-VSA2000B). The results are presented in Table 5. HPAM is sheared in the Mixing Speed Governor at 3600 RPM for 20 s, and the viscosity retention rate is 64.62%. The viscosity retention rate of HPAM is further reduced with increasing shear strength. HBPSs hyperbranched copolymer show higher viscosity retention rate and shear-resistance compared with HPAM in similar conditions. One of the main reasons is due to the linear structure of HPAM. However, the hyperbranched HBPSs copolymer can cut away a portion molecular chains, and its molecular weight and hydrodynamic radius hardly change due to the multi-side-chains structure of hyperbranched copolymer. The relatively minor effect of shear-thinning on the viscosity of HBPSs copolymer causes a higher viscosity retention rate compared with HPAM. The shear resistant capability of HBPS-2 is slightly stronger than HBPS-1 because it is compounded by a higher degree of modification in the nano-SiO_2_ and contains shorter branch chains. The destruction of HBPS-2 molecular chains, the change in molecular weight and the hydrodynamic radius are also less significant under the same shear conditions. Therefore, HBPS-2 has higher viscosity retention rate compared with HBPS-1.

### 3.5. Viscoelasticity of HBPS Copolymer

**Figure 7:**
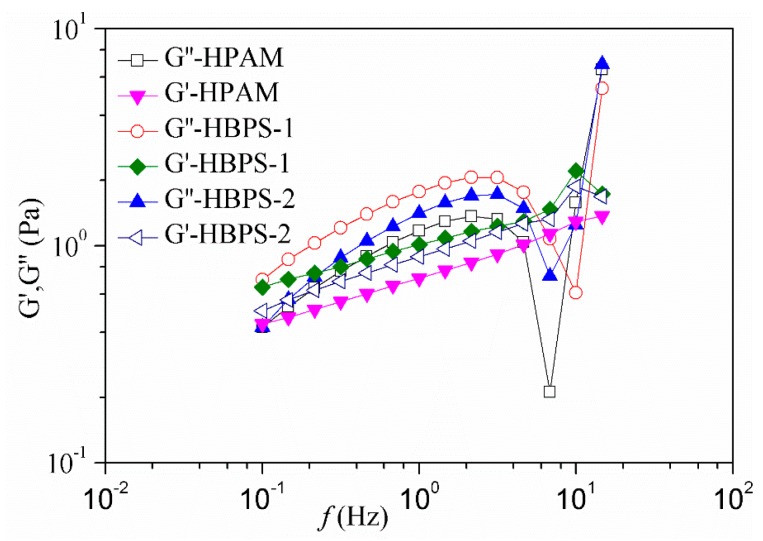
Viscoelastic behavior of polymer solutions.

In this section, the relationship between elasticity modulus (G’) and viscous modulus (G’’) of copolymers and the scanning frequency was measured. The curves obtained for HBPS have similar profiles as those for linear polymer solutions, in which viscoelasticity was governed by chain entanglement. They were characterized at low frequency (0.1~5.0 Hz) and high frequency (5.0~10.0 Hz) by the slopes of G’ and G’’. At low frequency, the values of G’ are lower than that of G’’, and this indicates that the elastic modulus is dominant. However at high frequency, the values of G’’ are lower that of G’, and this indicates that the viscous modulus is dominant. Under the same conditions, the viscous modulus and elastic modulus of HBPS-1 are greater than those of the modulus for HBPS-2 and HPAM. Preliminary analysis suggests that the molecular weight and hydrodynamic radius of HPAM are higher than hyperbranched HBPSs copolymers. HPAM is a linear polymer and begins to coil at 70 °C; therefore, its viscous modulus and elastic modulus are significantly lower comparatively. The viscosity modulus and elastic modulus of the HBPS-2 solution are lower than those of HBPS-1. The results are shown in Figure 7.

### 3.6. Feasibility of HBPS in Low-Mid Permeability Reservoirs

#### 3.6.1. Injectivity and Mobility Control Ability

**Figure 8:**
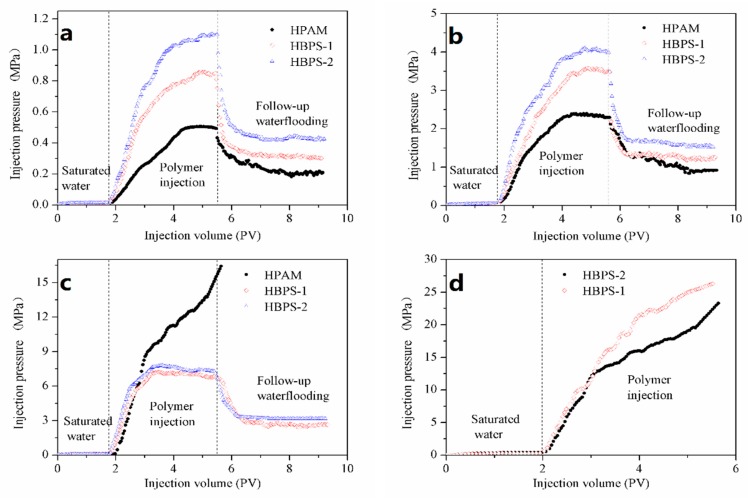
Flooding pressure curves of the HPAM, HBPS-1 and HBPS-2 solution at (**a**) 130 × 10^−3^ μm^2^, (**b**) 90 × 10^−3^ μm^2^, (**c**) 50 × 10^−3^ μm^2^ and (**d**) 10 × 10^−3^ μm^2^.

A prerequisite condition of a polymer as an EOR chemical agent is that the molecular dimensions must have good compatibility with the formation’s pore throat size. The flow characteristic curves of HBPS-1, HBPS-2 and HPAM under various permeabilities are presented in Figure 8. As can be seen from Figure 8, HBPS-1 and HBPS-2 present excellent injection performance at the permeability from 50 × 10^−3^ to 130 × 10^−3^ μm^2^. When the permeability decreases to 10 × 10^−3^ μm^2^, the injection pressure of the HBPS solution increases, and that implies that it has an inferior injectivity in porous media whose pore throat diameter is lower than 10 × 10^−3^ μm^2^. HPAM display possible injection performance at the permeability of approximately 90 × 10^−3^ μm^2^ and 130 × 10^−3^ μm^2^. However, the injection pressure of HPAM solution increases, which implies an inferior injectivity at the porous medium below 50 × 10^−3^ μm^2^. These findings confirm the conclusion that the diameters of HBPSs are smaller than the HPAM diameter. Furthermore, the pressure curve observed in Figure 8a–c proves that hyperbranched copolymers have better viscoelasticity than HPAM, and it can reduce water permeability by adsorbing and accumulating on the surface of rock [11]. When the displacing fluid enters the deep end of the reservoirs and the injection pressure rises to a threshold value, the hyperbranched polymer would make a breakthrough from the pore throats due to the good flexibility and viscoelasticity under the tensile force, and thus immensely accelerate the oil displacement.

The RF and RRF of HBPS-1, HBPS-2 and HPAM in the sand pack model are shown in Table 6. Under the same conditions, HBPSs can achieve a much higher RF and RRF in comparison with HPAM due to more branched chains and a higher viscosity. The results indicate that the HBPSs solution shows a stronger mobility control capability for enhanced oil recovery. On the other hand, the mobility control performance of the HBPS-2 copolymer is slightly stronger than HBPS-1. HBPS-2 has higher RF and RRF compared with HBPS-2 because it feels the effect of shearing less and is more readily adsorbed on the formation rock surface.

#### 3.6.2. EOR Ability

**Figure 9:**
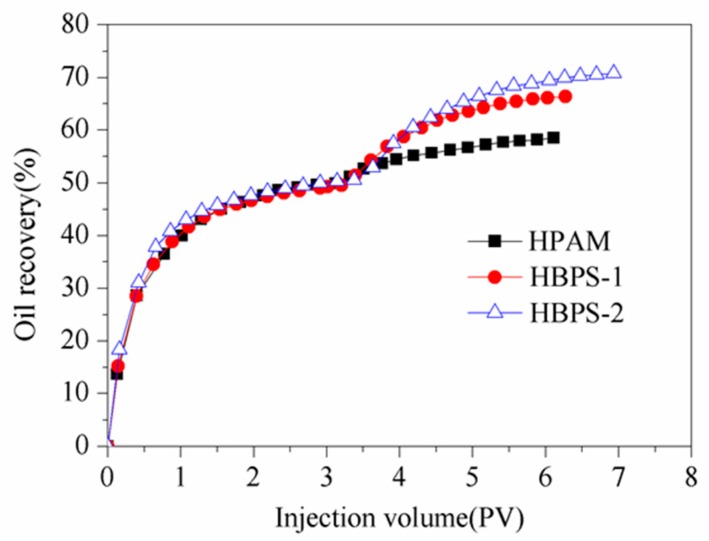
EOR ability for HPAM, HBPS-1 and HBPS-2.

Enhancing oil recovery by using HPAM, HBPS-1 and HBPS-2 was evaluated in a sand pack model with a permeability of 70 × 10^−3^ μm^2^. The results are shown in Figure 9 and Table 7. Following a 98.0% water cut after water flooding, 0.3 PV of polymer and extended water flooding can further increase the oil recovery by 16.25% (HBPS-1) and 18.74% (HBPS-2) in comparison to 8.12% oil recovery when using HPAM. The results indicate that due to its favorable shearing resistance, viscoelasticity and mobility control performances, the hyperbranched polymer demonstrates better EOR capability when compared with linear polymer in the low-mid permeability reservoirs.

## 4. Conclusions

Novel water soluble modified nano-SiO_2_ hyperbranched copolymers (HBPSs) were synthesized through an in situ free radical polymerization reaction. HBPSs showed extremely good mechanical and physical performance when compared with HPAM, especially for shear resistance and viscoelasticity. The injectivity and mobility control tests showed that HBPSs copolymer solutions displayed excellent injectivity performance and established a higher resistance factor and residual resistance factor in comparison to a HPAM solution. Compared with linear HPAM, the EOR value of hyperbranched copolymers was higher at similar permeability conditions. The development of new hyperbranched polymers provides an alternative approach to improve the recovery issues facing low-mid permeability reservoirs, and mitigates the influences of high-temperature and strong-shearing during the regular polymer flooding process.

## Figures and Tables

**Table 1:** Reservoir classification.

Level of Reservoirs	Permeability (10^−3^ µm^2^)	Permeability Category
Poor	<1	Tight
Fair	1~10	Low
Moderate	10~50	Medium
Good	50~250	Medium
Very good	>250	High

**Table 2:** Copolymerization conditions of HBPSs.

	Total *	AA	AM	NSIO	Initiator
Concentration (wt.%)	26.0	7.6	17.7	0.5	0.2

* Note: AA + AM + NSIO + Initiator = 26%.

**Table 3:** Ionic composition of the brine.

Ions	[Na^+^+K^+^]	[Ca^2+^]	[Mg^2+^]	[HCO_3_^−^]	[SO_4_^2−^]	[Cl^−^]
Content (mg/L)	2049	80	28	400	90	3080

**Table 4:** Structural Parameters of HPAM, HBPS-1 and HBPS-2.

Polymer	*M_W_* (10^7^ g/mol)	*R_g_* (nm)	*R_h_* (nm)	A (nm (mol/g) ^1/2^)
HPAM	1.24	129	315	0.090
HBPS-1	1.10	117	196	0.086
HBPS-2	1.02	96	158	0.074

**Table 5:** Viscosity retention rate of polymer solution after shearing.

Polymer *	$\eta_{{}_{b}}$ (mPa·s)	3600 RPM *t*_shearing_ = 20 s		7200 RPM *t*_shearing_ = 20 s		11500 RPM *t*_shearing_ = 20 s	
		$\eta_{a}\;(\mathrm{mPa}\cdot s)$	$\eta_{a}$ $/\eta_{{}_{b}}\;(\%)$	(mPa·s)	$\eta_{a}$ $/\eta_{{}_{b}}\;(\%)$	$\eta_{a}\;(\mathrm{mPa}\cdot s)$	$\eta_{a}$ $/\eta_{{}_{b}}\;(\%)$
HPAM	6.5	4.2	64.62	3.5	53.85	2.8	43.08
HBPS-1	8.1	7.1	87.65	6.8	83.95	5.9	72.84
HBPS-2	7.7	6.9	89.61	6.5	84.42	6.0	77.92

* Note: $\eta_{{}_{b}}$ is the polymer viscosity before shearing, $\eta_{a}$ is the polymer viscosity after shearing and $\eta_{a}$/$\eta_{{}_{b}}$ is the viscosity retention rate.

**Table 6:** RF and RRF of HPAM, HBPS-1 and HBPS-2.

Polymer	Permeability (× 10^−3^ μm^2^)	Stable Pressure (MPa)	Porosity (%)	RF	RRF
HPAM	47.76	/	20.88	/	/
HBPS-1	49.89	0.0851	21.61	80.19	31.12
HBPS-2	52.62	0.0807	21.85	90.72	39.11
HPAM	87.55	0.0481	23.79	47.40	18.88
HBPS-1	93.12	0.0456	24.67	76.45	27.67
HBPS-2	91.32	0.0465	24.31	85.73	32.73
HPAM	135.98	0.0110	28.23	41.74	17.41
HBPS-1	132.92	0.0129	27.98	66.01	23.47
HBPS-2	128.12	0.0151	27.42	72.30	28.07

**Table 7:** EOR of HPAM, HBPS-1 and HBPS-2.

Samples	Polymer	Permeability (× 10^−3^ μm^2^)	E_T_ (%)	E_W_ (%)	EOR (%)
#1	HPAM	74.89	54.30	46.18	8.12
#2	HBPS-1	78.49	64.11	47.86	16.25
#3	HBPS-2	81.18	68.36	49.62	18.74

## Supplementary Materials

The following are available online at https://www.mdpi.com/2073-4360/11/9/1483/s1, Figure S1: Flow-process diagram of CH_3_MgCl titration, Table S1: Quantity of hydroxyl groups by CH_3_MgCl titration, Table S2: Content of surface amino groups after grating KH540 to nano-SiO_2_, Figure S2: Effect of KH540 dosage on surface modification degree of nano-SiO_2_.
