# Supplementary material for: Feasibility Study of Applying Modified Nano-SiO2 Hyperbranched Copolymers for Enhanced Oil Recovery in Low-Mid Permeability Reservoirs
Source: Polymers (Basel). 2019 Sep 11;11(9):1483. doi: 10.3390/polym11091483 (PMC6780960; doi:10.3390/polym11091483)
Supplement: Supplementary file 1 [file polymers-11-01483-s001.pdf]

polymers

Feasibility Study of Applying Modified Nano-SiO_2 Hyperbranched Copolymers for Enhanced Oil Recovery in Low-Mid Permeability Reservoirs

Nanjun Lai [1,2,3,*], Lei Tang [1], Na Jia [4], Dongyu Qiao [5], Jianlin Chen [6], Yong Wang [6] and Xubin Zhao [6]

[1] School of Chemistry and Chemical Engineering of Southwest Petroleum University, Chengdu 610500, China; 201811000045@stu.swpu.edu.cn
[2] State Key Laboratory of Oil and Gas Reservoir Geology and Exploitation, Chengdu University of Technology, Chengdu 610500, China
[3] State Key Laboratory of Molecular Engineering of Polymer, Fudan University, Shanghai 200433, China
[4] Faculty of Engineering and Applied Science, University of Regina, Regina, SK S4S 0A2, Canada; Na.Jia@uregina.ca
[5] Engineer Technology Research Institute, CNPC Xibu Drilling Engineering Company Limited, Urumqi 830001, China; xjqdy@cnpc.com.cn
[6] CNPC Xinjiang Oilfield Company, Karamay 834000, China; 201811000047@stu.swpu.edu.cn (J.C.); lainanjun@swpu.edu.cn (Y.W.); lainanjun@163.com (X.Z.)
[*] Correspondence: lainanjun@126.com; Tel: +86-13094484238

Received: 8 August 2019; Accepted: 9 September 2019; Published: date

The Method of Determining the Degree of Modification Nano-SiO_2. Determination of Quantity of Surface Hydroxyl Groups of Nano-SiO_2.The Grignard reagent (CH_3MgCl) was utilized to react with surface active hydrogen of nano-SiO_2 to release CH_4 gas, and consequently the content of surface silicon hydroxyl groups could be determined, with the reaction scheme as follows[1-3]. Firstly, hoping to remove the bound water, nano-SiO_2 was activated in an oven at 110 °C for 12 h. Secondly, 4.0 ml CH_3MgCl was added in 250 ml pressure equalizing addition funnel. 1.0 g nano-SiO_2 and 100ml toluene were added in 250 ml conical flask. Finally, the flow-process diagram of reaction was set up as follow in Figure S1.

$$SiOH + CH_3MgCl \longrightarrow SiOMgCl + CH_4 \uparrow$$

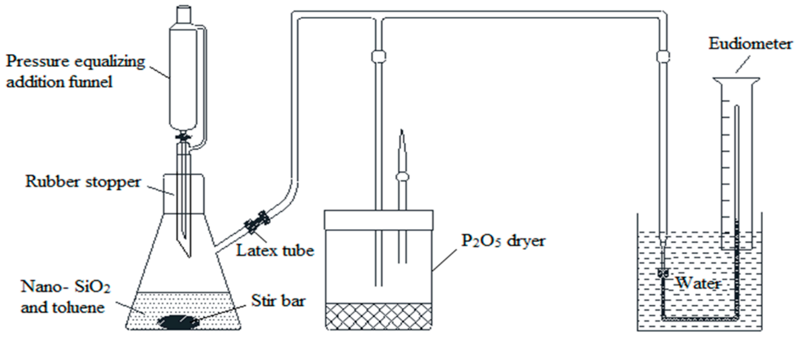

Figure S1. Flow-process diagram of CH_3MgCl titration.

To calculate quantity of silicon hydroxyl groups by equation S1:

$$N = \frac{P \times (V - V_K) \times N_A}{R \times T \times S \times m} \quad (S1)$$

Where, N—Quantity of surface hydroxy groups, number/unit, P—Atmospheric pressure (Pa); V—The Volume of CH_4 in eudiometer (m^3), V_K—Blank experiment CH_4 volume (m^3); N_A—Avogadro

constant, R—Gas constant; *T*—Experiment temperature (K), *S*—Specific surface area of sample (m^2/g); *m*—Sample mass (g).

The results of quantity of surface hydroxy groups of nano-SiO_2 by CH_3MgCl titration are as shown in Table S1.

Table S1. Quantity of hydroxyl groups by CH_3MgCl titration.

No.	Mass/g	Gas volume/ml	Hydroxyl groups/ mmol/g
1	1.0633	31.4	1.1491
2	1.0007	29.8	1.1587
3	1.0880	32.3	1.1552
Average	1.0507	31.2	1.1543

The experimental results show that the content of surface hydroxy groups of nano-SiO_2 is within the range of 1.149~1.159 mmol/g.

Determination of quantity of monomer amino groups of modified nano-SiO_2. Prepare and calibrate about 0.100 mo1/L of hydrochloric acid-ethanol standard solution[4-7], and dilute the standard solution 10 times for standby use. Weigh a certain amount of modified nano-SiO_2 samples (Firstly, 5.0 g nano-SiO_2 and 100.0 ml toluene solvent were added in 250 ml round flask. Then 2.0 g KH540 was added slowly into the round flask, and the mixture was reacted under magnetic mixing at 80 °C for 12 h. After that, ethanol was utilized to wash the unreacted KH540 and toluene solvent in rotary steaming instrument for multiple times.), and dispersed the samples in ethanol. Add indicator thymol blue after the samples disperse evenly, and titrate it with hydrochloric acid-ethanol standard solution. The end point of titration reaches when the system turns into pink from light yellow, and does not fade in 30 s. The amino value of nano-SiO_2 surface is calculated in equation S2:

$$C_N = \frac{c \times (V - V_0)}{m} \times 100 \qquad \text{(S2)}$$

Where, *c* is the concentration of hydrochloric acid—ethanol solution (mol/L); *m* is the mass of modified nano-SiO_2 (g); *V* is the reaction of amino to the volume of hydrochloric acid—ethanol solution (mL), V_0 is the volume of hydrochloric acid—ethanol solution of a blank testing (mL).

Table S2. Content of surface amino groups after grating KH540 to nano-SiO_2

No.	Mass (g)	V_{HCl}(ml)	V_0(ml)	C_{HCl}(mol/L)	Amino value (mmol/g)
No.1	0.4662	20.42	0.02	0.008818	0.3859
No.2	0.4864	21.28	0.02	0.008818	0.3854
No.3	0.4626	20.14	0.02	0.008818	0.3835
Average	0.4717	20.61	0.02	0.008818	0.3849

As can be seen from Table S2, the content of surface amino groups of modified nano-SiO_2 is about 0.383–0.386 mmol/g. According to the reaction principle, 1 mol KH540 can react with 3 mol silicon hydroxyl groups, and is less than one third of surface hydroxy groups of nano-SiO_2, which shows that the modification is feasible.

Effect of Feeding on Modification Degree. Weigh 1.0 g nano-SiO_2 (dry it for 12 h at 110 °C before using it), add different amounts of KH540 and 16.0 ml toluene, and let them react for 12 h at 80 °C. After reaction, get the white powder modified nano-SiO_2 after drying the reaction product for 24 h at 110 °C in vacuum after utilizing ethanol to wash away the unreacted KH540 and toluene for multiple times. Utilize the hydrochloric acid—ethanol non-aqueous titration to determine the content of amino proups of the product surface, and calculate the modification degree of surface hydroxy groups of nano-SiO_2 with the results as shown in Figure S2.

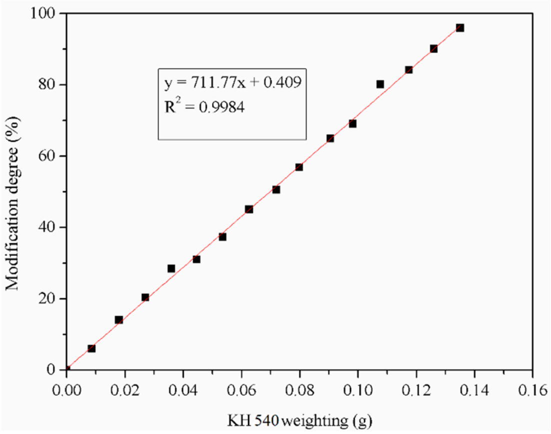

Figure S2. Effect of KH540 dosage on surface modification degree of nano-SiO_2.

As can be seen from Figure S2, the content of surface amino groups of modified nano-SiO_2 can be controlled by the control of KH540 dosage. With the increase of KH540 dosage, the content of amino groups gradually increases, and the modification degree increases correspondingly. When the KH540 dosage is 0.0365 g, the content of surface amino groups of modified nano-SiO_2 is 0.1012 mmol/g, and the modification degree is 26 %. When the KH540 dosage is 0.0672 g, the content of surface amino groups of modified nano-SiO_2 is 0.1848 mmol/g, and the modification degree is 48 %. The above results show that the modification of surface hydroxy groups can be achieved through control the modification reaction conditions.

References

1. Rong M, Zhang M, Zheng Y, Friedrich, K. Improvement of tensile properties of nano-SiO_2/PP composites in relation to percolation mechanism. *Polymer* **2001**, 42, 3301-3304.
2. Sun, Y., Zhang, Z., Wong, C. Study on mono-dispersed nano-size silica by surface modification for underfill applications. *J. Colloid Interf. Sci*. **2005**, 292, 436-444.
3. Espiard, P., Guyot, A. Poly(ethyl acrylate) latexes encapsulating nanoparticles of silica: 2. Grafting process onto silica. *Polymer* **1995**, 36, 4391-4395.
4. Walcarius, A., Etienne, M., Bessière, J. Rate of Access to the Binding Sites in Organically Modified Silicates. 1. Amorphous Silica Gels Grafted with Amine or Thiol Groups. *Chem. Mater*, **2002**, *14*, 2757–2766.
5. Blaaderen, A.V., Vrij, A. Synthesis and Characterization of Monodisperse Colloidal Organo-silica Spheres. J. *Colloid Interf. Sci*. **1993**, *156*, 1–18.
6. Etienne, M., Walcarius, A. Analytical investigation of the chemical reactivity and stability of amine-functionalized silica in aqueous medium. *Talanta* **2003**, *59*, 1173–1188.
7. Gimpel, M., Unge, K. Hydrolytically stable chemically bonded silica supports with metal complexating ligands: Synthesis, characterization and use in high-performance ligand-exchange chromatography. *Chromatographia* **1982**, *16*, 117–125.
